# Formation of 5-methyl-2-furanmethanol which is a caramel flavor compound in the glutamic acid-glucose Maillard reaction: A ReaxFF-DFT study

**DOI:** 10.1016/j.crfs.2026.101454

**Published:** 2026-05-26

**Authors:** Xin Pan, Chao-Kun Wei, Wen-Li Wang, Ya-Nan Weng, An-Ran Zheng, Lian-Qiang Sun, Chun-Mei Yang, Yuan Liu

**Affiliations:** a School of Food Science and Engineering, Ningxia University, Yinchuan, 750021, People's Republic of China; b School of Biological Science and Engineering, North Minzu University, Yinchuan, 750021, People's Republic of China; c School of Agriculture & Biology, Shanghai Jiao Tong University, Shanghai, 200240, People's Republic of China; d Ningxia Ningyang Food Co., Ltd., Wuzhong, 751100, People's Republic of China

**Keywords:** Maillard reaction, Caramel flavor, 5-Methyl-2-furanmethanol, ReaxFF MD, DFT

## Abstract

We identified 5-methyl-2-furanmethanol as a key caramel flavor compound in the glutamic acid-glucose Maillard reaction using gas chromatography-olfactometry-mass spectrometry (GC-O-MS) and elucidated its formation pathway using reaction molecular dynamics (ReaxFF MD) and density functional theory (DFT). The pathway was validated by pyrolysis-gas chromatography-mass spectrometry (Py-GC-MS). Results indicate that glutamic acid reacts with glucose to form 1-deoxyglucosone, which undergoes homolytic cleavage to generate the 3-formyl-1,2,3-trihydroxypropyl radical and the 1,2-dihydroxyethyl radical. These findings align with GC-O-MS trends, validating the proposed synthesis pathway of 5-methyl-2-furanmethanol.This study provides a reference for future research on caramel flavor compounds generated by the Maillard reaction.

## Highlights

• GC-O-MS identified 5-methyl-2-furanmethanol as the key caramel flavor compound.
• Integrated ReaxFF pathways and DFT revealed 5-methyl-2-furanmethanol formation.
• This compound is formed primarily through addition, deprotonation, and isomerization.

## Graphical abstract

**Figure:**
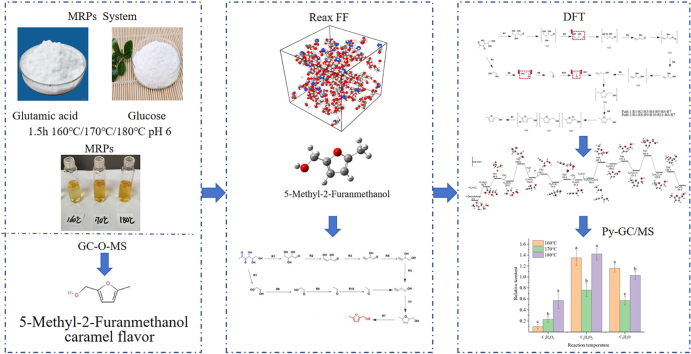

## 1 Introduction

The Maillard reaction is a type of non-enzymatic browning that refers to the chemical reaction between amino compounds and carbonyl compounds. Factors influencing the Maillard reaction include temperature, time, pH, and reducing sugars. This paper focuses on the effect of temperature on the Maillard reaction (Chen et al., 2018, 2019; Han et al., 2023; Hu et al., 2022). The Maillard reaction is widely used in thermal food processing as a primary source of flavor compounds (Huang et al., 2025). Maillard reaction products (MRPs) are recognized as precursors to processing flavors, capable of undergoing further cleavage reactions upon heating. These reactions generate numerous volatile flavor compounds that contribute significant aroma and taste characteristics to food products (Zhang et al., 2021; Gao et al., 2020). Consequently, MRPs have garnered considerable interest in the food industry as potential flavor enhancers and food additives (Huang et al., 2023). Glutamic acid and glucose are common Maillard substrates in food systems, producing aroma-active MRPs that contribute caramel, roasted, and nutty notes (Löliger, 2000). As a result, numerous studies have focused on producing MRPs using glutamic acid and glucose as substrates. These products are particularly effective in generating caramel or burnt flavor notes, thereby enhancing the development of richer and more complex aromas in baked goods and meat products (Chen et al., 2024). Therefore, in-depth study of the Maillard reaction mechanism of glutamic acid-glucose and its flavor formation pathway has important theoretical significance and application value for the development of new flavor additives and the improvement of food quality.

MRPs derived from glutamic acid are widely employed to enhance and modify the caramel flavor profiles of food products (Xu et al., 2019). For instance, the addition of glutamic acid to a cysteine-xylose-glutamic acid reaction system was shown to shift the resulting flavor from grilled notes toward a broth-like character (Zheng et al., 2023). Similarly, incorporating soy protein isolate into bread significantly increased the content of furfural, a compound generated through the Maillard reaction between reducing sugars and glutamic acid that imparts a roasted almond-like note compared to conventional bread (Xu et al., 2019). Studies on the thermal reaction of glucose with glycyl-L-glutamine have demonstrated that the heating temperature substantially influences flavor formation in the resulting products (Agcam, 2022). Furthermore, the thermal degradation of Amadori compounds promotes the formation of pyrazines and furans, which are key contributors to nutty, fatty, and baked flavor notes (Xia et al., 2023). It has also been shown that Amadori compounds derived from glutamic acid and xylose, as well as unreacted glutamic acid-xylose mixtures, rapidly produce abundant volatile flavor compounds with biscuit-like roasted aromas, indicating their potential as flavor enhancers in baked goods (Xu et al., 2019). Moreover, MRPs from wolfberry seed meal were identified as sources of meat-like flavor substances and precursor peptides. Notable sulfur-containing compounds such as 3-methyl-2-thiophenecarboxaldehyde, 2-furfuryl mercaptan, and 2-acetylfuran were detected at concentrations as high as 1110.0 ng/g (Shang et al., 2025). Such Maillard-derived caramel flavorings are highly valued by consumers because the diverse key volatile compounds contribute rich and complex aromas, endowing these seasonings with unique sensory qualities (Huang et al., 2024a, Huang et al., 2024b). Therefore, incorporating MRPs from the glutamic acid-glucose reaction into food systems can significantly improve both flavor and color. Investigating the aromatic characteristics of these compounds is of considerable importance for the optimization and enhancement of food flavor profiles (Gao et al., 2020; Zhang et al., 2021). Furan compounds are the key volatile compounds in the Maillard reaction (Du et al., 2023). 5-Methyl-2-furanmethanol has a characteristic caramel, bread-like aroma and serves as a key aroma contributor in baked goods (Lasekan et al., 2021). The concentration of 5-methyl-2-furanmethanol increases significantly after fermentation, while typical “yeasty” compounds (propionic acid, butyric acid) are completely removed. This increase in the compound is one of the positive indicators of successful fermentation (Ma et al., 2021). 5-Methyl-2-furanmethanol is an important caramel-flavor volatile compound in glucose-glutamate Maillard reaction products and is recognized as a key aroma contributor in various food matrices. It is an important contributor in various hot-processed foods. Understanding its formation mechanism will help the food industry to enhance or optimize the caramel flavor of food by accurately controlling raw materials and processing conditions and improve the overall palatability and market appeal of condiment products. Therefore, studying the thermal decomposition mechanism of 5-methyl-2-furanmethanol during the Maillard reaction is significant for understanding volatile formation and the caramel aroma in foods.

Currently, most research on the Maillard reaction focuses on the content and types of volatile substances (Gao et al., 2025; Pan et al., 2025). However, these studies cannot explore the reaction mechanism from a microscopic perspective. Therefore, we used ReaxFF, a computational method that can systematically study reaction pathways and analyze chemical reactions from a microscopic angle, to investigate the glucose-glutamate reaction system (Chen et al., 2023; Schmalz et al., 2024). ReaxFF is a reactive force field (Zeng et al., 2021). With the continuous improvement in computational power in recent years and an in-depth understanding of reaction mechanisms, ReaxFF plays an important role in predicting material properties, optimizing reaction pathways, and exploring chemical reaction mechanisms (Lian et al., 2025; Ma et al., 2025). The application of Reax FF MD in food science research is expected to provide an in-depth mechanistic understanding of key processes such as the Maillard reaction, lipid oxidation, and protein modification, thereby offering a theoretical basis for flavor regulation, nutrient retention, and safety control (Gao et al., 2024). Using ReaxFF MD, researchers simulated structural changes in food enzymes under high-temperature and high-pressure conditions, providing insights into their functional adaptability in complex industrial environments (Liu et al., 2025). DFT is one of the most widely used methods in theoretical chemistry (Mijajlović et al., 2024). It offers valuable insights into chemical reactions by enabling the calculation of molecular structures, reaction parameters, transition states, and products (Yang et al., 2022). In previous studies, DFT has been employed to perform detailed calculations of Maillard reaction processes, leading to an improved molecular-level understanding of the reaction mechanisms and supporting strategies to control the formation of certain harmful byproducts (Gao et al., 2023). Additionally, DFT can identify active sites of functional groups and determine reaction activation energies (Lu et al., 2025; Xiao et al., 2023). DFT simulations were used to investigate the interactions between nano-chemical response materials (CRMs) and volatile compounds in tea. The results indicate that CRMs sensitive to volatile alcohol exhibit a smaller energy gap (Kang et al., 2022). Using DFT, we investigated the molecular-level evidence supporting the use of oligo-fucoidan sulfate as a multifunctional natural additive in cheese. It can regulate cheese quality by encapsulating volatile flavor compounds (butyric acid, diacetyl) for controlled release, buffering pH through reversible binding with lactic acid, and protecting linoleic acid from oxidation (Anjum V et al., 2025). Thus, applying DFT in flavor chemistry can thus provide a theoretical foundation for elucidating the molecular formation mechanisms of food flavor compounds.

Although 5-methyl-2-furanmethanol is a key volatile substance in the Maillard reaction, but its formation path has not been systematically studied. Based on this, present study first conducted GC-O-MS analysis of volatile compounds in the glutamic acid-glucose Maillard reaction system. The volatile compound 5-methyl-2-furanmethanol, which contributes to a caramel aroma, was identified. In this study, the reaction path of 5-methyl-2-furanmethanol was obtained by using Reax FF and DFT. The basic reaction pathway of 5-methyl-2-furanmethanol was studied by Reax FF and use DFT to supplement the path. DFT was used to study the vibrational direction of the transition states, and Gaussian software was used to calculate the transition states in the reaction process of volatile compounds. The reactants, intermediates, transition states, and products were comprehensively optimized. Furthermore, the energy levels of the highest occupied and lowest unoccupied molecular orbitals (HOMO and LUMO) were calculated to evaluate the reaction stability. The computational results were further corroborated experimentally by pyrolysis–gas chromatography–mass spectrometry (Py–GC–MS). It provides a theoretical basis for further study of 5-methyl-2-furanmethanol and provides a theoretical basis for further study of the mechanism of MRPs caramel aroma.

## 2 Materials and methods

### 2.1 Materials

*L*-glutamic acid (98%, analytical grade), *D*-anhydrous glucose (98%, analytical grade), 5-Methyl-2-Furanmethanol (97%, analytical grade) were purchased from Shanghai Maclean's Biochemical Technology Co. Glycerol (analytical grade) was purchased from Tianjin Damao Chemical Reagents Partnership Enterprise; Ltd. Sodium dihydrogen phosphate (analytical grade), disodium hydrogen phosphate (analytical grade), methanol (Chromatography grade) were purchased from Beijing Chemical Reagent Company. The 1,2-dichlorobenzene reference standard (Chromatography grade) and n-alkane (C_7_∼C_30_) were purchased from Sigma-Aldrich (USA).

### 2.2 Preparation of the glutamic-containing Maillard system

Glucose (0.3 g) and glutamic acid (0.2 g) were weighed and dissolved in 10.0 mL of phosphate buffer solution (0.2 mol/L, pH 6), mixed well, transferred to a 20.0 mL pressure-resistant flask, and reacted at 160 °C, 170 °C, and 180 °C for 1.5 h, respectively. The prepared Maillard reaction products were pre-frozen at −80 °C for 24 h, then freeze-dried and stored at 4 °C until use.

### 2.3 Determination of the odor threshold of 5-methyl-2-furanmethanol

Sensory threshold determination strictly follows ISO 13301:2018 standards. A caramel aroma is a complex flavor profile characterized primarily by notes of caramel and nuts, accompanied by a slight bitterness, resulting from the formation of volatile compounds such as pyrazines and furans during the Maillard reaction (along with some caramelization) as food is heated. High-purity 5-methyl-2-furanmethanol was dissolved in a deodorized neutral medium, and a series of concentration gradient samples was prepared using a stepwise geometric dilution method (dilution factor of 3). Screened and trained evaluators conducted progressive testing in a standardized sensory laboratory using the three-point forced-choice (3-AFC) method. Starting at low concentrations, evaluators identified the outlier among three samples (one containing the target compound, two blanks). All evaluator endpoint data were analyzed using probability unit analysis or logistic regression to fit dose-response curves. The concentration corresponding to a 50% probability of correct detection was designated as the detection threshold. Statistical software was employed to calculate repeatable population threshold results.

### 2.4 GC-O-MS analysis

GC-O-MS analyses were performed on a DB-WAX capillary column (30 m × 0.25 mm × 0.25 μm). Helium was used as the carrier gas at a constant flow of 1.0 mL/min. Injectors operated in split less mode at 250 °C. The oven temperature program was as follows: initial temperature was held at 40 °C for 2 min, increased to 80 °C at a rate of 3 °C/min and held for 3 min, then ramped to 120 °C at 4 °C/min and held for 2 min, and finally raised to 230 °C at 10 °C/min. The mass spectrometer operated in EI mode at 70 eV; ion source 230 °C and quadrupole 150 °C. Data were acquired in full scan (m/z 40–450). Compound identification relied on MS matching against the NIST17 library, with a similarity threshold >80%. An olfactometry port was coupled with the GC-MS system for sensory evaluation. Six evaluators (three male, three females; average age 24; all with ≥1-year sensory experience) independently assessed each sample after a two-week training focused on caramel-related aromas. Each sample was analyzed six times, one evaluator per run. An aroma-active compound was considered confirmed if detected in ≥3 of the 6 runs (detection frequency DF ≥ 3).

### 2.5 Simulation method of Reax FF

**Fig. 1:**
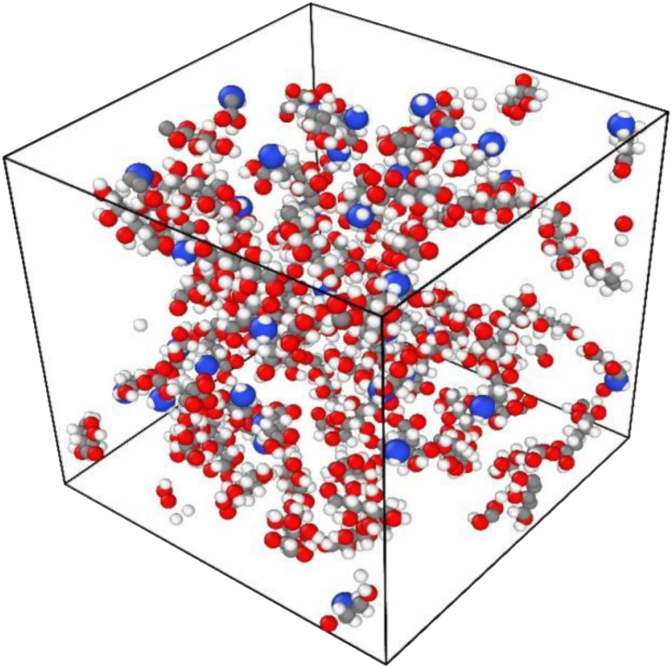
The 3D model of the multi-molecular mixture of glutamic acid-glucose Maillard product.Among them, the gray, white, blue and red subtables represent C, H, N and O atoms.

The single-molecule models of glutamate and glucose were constructed in Materials studio, geometrically optimized in the Dmol3 module, and the multimolecular mixture model was constructed in the AC module. The density of the model is 0.2 g/cm^3^, and the size of the model box is (49.9831∗49.9831∗49.9831 Å3). In this study, a 3D model of the multimolecular mixture of glutamate-glucose MRPs was selected as shown in Fig. 1, where the gray, white, blue and red subtables represent the C, H, N and O atoms. The total simulation time is set to 2 ns, the simulation time step is 0.2 fs, and the output interval of the analog trajectory is 2 ps.

### 2.6 Quantum chemistry calculations

Geometry optimizations and transition state searches were performed at the B3LYP-D3/6-311G+(d,p) level using Gaussian 16. Vibrational frequency analyses were conducted at the same level to verify the nature of stationary points (minima with all real frequencies; transition states with one imaginary frequency).Single-point energies and refined energetics were subsequently computed on the optimized structures at the B3LYP-D3/6-311G+(d,p) level (including zero-point energy corrections from the frequency calculations). All energies are reported in kcal mol^−1^. For comparison and robustness, representative key structures (reactants, intermediates, transition states, and products) were also evaluated at a higher basis set, using B3LYP-D3, and results were found to be consistent within the expected computational uncertainty.

### 2.7 Py-GC-MS analysis

The pyrolysis (CDS6200,American CDS Company) process was conducted under the following temperature program: the initial temperature was held at 25 °C for 10 min, increased to 72 °C at 1 °C/min, then ramped to 280 °C at 5 °C/min and held for 5 min, and finally raised to 750 °C at 20 °C/s with a final hold time of 10 s. The resulting pyrolysis products were directly transferred to the GC-MS (LCQDecaXPMax, Thermo Fisher Scientific Inc) system for analysis.Gas chromatographic separation was performed on a DB-5MS fused-silica capillary column (30 m × 0.25 mm × 0.25 μm). The injector temperature was maintained at 280 °C. A 1 μL sample was injected in split mode with a split ratio of 25:1. The oven temperature was programmed as follows: held at 50 °C for 2 min, then increased to 280 °C at a rate of 5 °C/min, and finally held at 280 °C for 20 min.Mass spectrometry was operated in electron ionization (EI) mode at 70 eV. The ion source and transfer line temperatures were set at 230 °C and 280 °C, respectively. The mass spectrometer was set to full-scan mode over a mass range of 30–550. Compound identification was achieved by comparing the acquired mass spectra with those in the NIST17 mass spectral library.

### 2.8 Statistical analysis of data

Statistical analysis of one-way ANOVA using SPSS Statistics 20.0 indicates a significance level of *p* < 0.05. All experimental data are expressed as mean ± standard deviation, with three replicates per group.

## 3 Results and discussion

### 3.1 Analysis of volatile components in glutamic acid-glucose Maillard reaction

Temperature is an important influencing factor of Maillard reaction. Increasing temperature is beneficial to the formation of caramel flavor compounds, but too high temperature will inhibit their formation (Pan et al., 2025). This study employed GC-O-MS to analyze volatile compounds from MRPs at three temperature conditions: 160 °C, 170 °C, and 180 °C The GC-O-MS identification results for volatile components are presented in Table 1. OAV is usually used to represent the contribution of aroma components to the overall flavor (OAV = substance content/substance threshold). OAV >1 indicates that the aroma component has a direct impact on the flavor of the substance. The larger the OAV value, the higher the contribution of the aroma component to the overall flavor. In view of the lack of threshold data for 5-methyl-2-furanmethanol in the literature, we used the three-point method to determine the threshold, and the determination result was 0.1 mg/kg. A total of 7 aroma substances that directly affected the flavor of glutamic acid-glucose MRPs were screened by threshold and OAV value calculated according to the formula. They are 2-acetylpyrazine, 2-pentylfuran, phenylethylaldehyde, 5-methyl-2-furanmethanol, phenylaldehyde, 1-octen-3-ol, acetylbenzone. Seven substances with OVA >1 were selected, as shown in Table 2. To regulate the quality of caramel flavor compounds produced by the glutamic acid-glucose Maillard reaction, this study utilized GC-O-MS analysis and odor activity value calculations to demonstrate that 5-methyl-2-furanmethanol generates a distinctive caramel aroma and serves as a key and pleasant pyrolytic flavor compound in thermally processed foods. Its odor threshold is as low as 0.10 mg/kg, indicating that this compound possesses extremely high flavor intensity and can be detected by the olfactory system even at very low concentrations. Although 5-methylfuranaldehyde is also a compound with a caramel aroma in this system, its OAV is relatively high, and its contribution to the caramel aroma is less significant than that of 5-methyl-2-furanmethanol. Furthermore, at all tested temperatures (160, 170, and 180 °C), the OAV of 5-methyl-2-furanmethanol remained consistently above 1 (ranging from 14.50 to 17.30) confirming that this compound is a key caramel flavor compound in this reaction system. Therefore, subsequent research will focus on 5-methyl-2-furanmethanol.

**Table 1:** Identification results of volatile components in glutamic acid-glucose Maillard reaction system by GC-O-MS.

Substance category	Compound Name	160 °C		170 °C		180 °C		Odor description^b^	RI^d^
		Relative content^a^	Odor intensity^c^	Relative content^a^	Odor intensity^c^	Relative content^a^	Odor intensity^c^		
Alcohols	1-Octen-3-ol	0.11 ± 0.04	-	3.11 ± 0.12	M	1.30 ± 0.06	W	Mushroom flavor	1790
	Erythritol	0.17 ± 0.06	-	2.15 ± 1.02	W	0.88 ± 0.01	W	Sweetness	1229
	Glycerol	23.75 ± 0.32	S	32.7 ± 0.95	S	21.30 ± 0.06	S	Sweetness	1115
	5-Methyl-2-Furanmethanol	1.73 ± 0.04	W	1.55 ± 0.01	W	1.45 ± 0.05	-	caramel aroma	1732
	1-Pentanol	0.55 ± 0.02	-	0.85 ± 0.05	1.5	0.44 ± 0.01	1.0	Fresh, woody notes	1457
	Sorbitol	0.01 ± 0.01	-	0.11 ± 0.01	W	0.07 ± 0.01	-	Sweetness	1752
Aldehydes	Aromatic aldehyde	0.15 ± 0.05	-	0.31 ± 0.03	W	0.22 ± 0.04	-	Citrus fragrance	1104
	Phenylaldehyde	9.82 ± 0.03	S	0.21 ± 0.02	-	8.47 ± 0.04	S	Smoky aroma	1040
	3-Furaldehyde	2.42 ± 0.05	W	0.12 ± 0.02	-	2.16 ± 0.03	W	Toasty aroma	831
	5-Methylfuranaldehyde	3.53 ± 0.07	M	3.30 ± 0.07	M	2.80 ± 0.14	M	Caramel flavor	920
	Phenylethylaldehyde	3.14 ± 0.04	M	4.39 ± 0.04	M	5.16 ± 0.03	S	Green, grassy notes	1081
	2,4-Dihydroxybut-3-enal	0.15 ± 0.09	-	0.55 ± 0.02	-	1.65 ± 0.02	W	Spicy flavor	1343
	Acetaldehyde	3.27 ± 0.01	M	2.05 ± 0.02	W	3.12 ± 0.07	W	Stimulating flavor	704
	2-Hydroxyacetaldehyde	3.56 ± 0.03	W	2.43 ± 0.02	W	4.01 ± 0.05	M	Nutty aroma	847
Furan derivatives	3,4-Epoxytetrahydrofuran	0.13 ± 0.05	-	0.41 ± 0.06	-	0.64 ± 0.02	W	Sweet aroma	2370
	2-Pentylfuran	2.55 ± 0.04	M	0.76 ± 0.03	W	3.83 ± 0.03	M	Baked flavor	990
Esters	1,3-Benzenediol	0.21 ± 0.03	-	0.51 ± 0.02	W	0.65 ± 0.03	W	Sweetness	1290
Ethers	Ethyl decanoate	0.15 ± 0.01	-	0.55 ± 0.01	W	0.21 ± 0.04	-	Coconut fragrance	1381
	Tetrahydro-2-furanacetic acid methyl ester	0.14 ± 0.03	-	0.31 ± 0.02	W	0.21 ± 0.04	-	Fruity notes	1016
	Ethyl acetate	0.19 ± 0.02	-	0.22 ± 0.06	W	0.24 ± 0.02	W	Waxy scent	2383
	Nonyl acetate	0.18 ± 0.03	W	0.12 ± 0.02	-	0.12 ± 0.03	-	Mushroom, gardenia fragrance	1282
Pyrazine	Ethyl stearate	2.6 ± 0.04	S	0.31 ± 0.03	-	3.16 ± 0.02	S	Slightly waxy	2177
	Diethyl phthalate	1.56 ± 0.01	W	3.20 ± 0.18	S	2.55 ± 0.02	M	Fragrant	1639
	Benzyl butyl ether	0.04 ± 0.02	-	0.35 ± 0.02	W	0.06 ± 0.04	-	Rose and stork's bill fragrance	1447
	2-Acetylpyrazine	0.28 ± 0.03	M	0.33 ± 0.04	S	0.14 ± 0.05	W	Roasted meat notes	1350
ketones	Phenylacetone	5.70 ± 0.05	W	8.30 ± 0.04	M	4.50 ± 0.01	W	Hawthorn flavor	1372
Acids	Lauric acid	19.49 ± 0.05	S	22.9 ± 0.57	S	23.48 ± 0.12	S	Fragrant laurel oil	1570

a Internal Standard Quantification: Add 2 μL of 1,2-dichlorobenzene (50 μg/mL in methanol) as an internal standard to each sample. Cv = $\frac{\text{Sv}}{\text{Si}}\times \text{Ci}$ (where Cv represents the concentration of the volatile compound and Ci represents the concentration of the internal standard; Sv represents the peak area of the volatile compound and Si corresponds to the peak area of the internal standard).

b The Odor characteristic of each volatile compound sensed on sniffer port during GC-O-MS experiments. The determination of odor was based on the detection frequency (DF ≥ 3).

c Odor intensity: “W” represents weak intensity, “M” represents moderate intensity, “S” represents strong intensity.

d RI, the retention index obtained by GC-O-MS (column: DB-wax,30 m × 250 μm,0.25 μm film thickness).

**Table 2:** The key volatile substances in the glutamic-glucose Maillard reaction system identified by GC-O-MS and OAV >1.

	Compound name	Odor description^a^	Threshold (mg/kg)	OAV^b^		
				160 °C	170 °C	170 °C
1	2-Acetylpyrazine	barbecue flavor	0.01	28.00	33.00	14.00
2	2-Pentylfuran	Earth, bean, fruit and vegetable flavors	0.10	25.50	7.60	38.30
3	Phenylethylaldehyde	honey fragrance	0.08	39.25	54.88	64.50
4	5-Methyl-2-furanmethanol	caramel flavor	0.10	17.30	15.50	14.50
5	Phenylaldehyde	floral flavor	0.97	5.88	8.56	4.64

a The Odor characteristic of each volatile compound sensed on sniffer port during GC-O-MS experiments.

b OAV = substance content/substance threshold. OAV ≥1 contributes more to the overall flavor, and the greater the OAV value of the volatiles, the greater the impact on the overall flav

### 3.2 The effect of temperature on the number of pyrolysis substances and the types of products

The aforementioned studies indicate that temperature has a significant effect on the volatile products of the Maillard reaction. In this study, the ReaxFF reaction field was employed to simulate high-temperature pyrolysis of the glutamic acid–glucose Maillard reaction system at 2700 K, 3000 K, and 3300 K, using temperature profiles that mirrored those observed in the experimental chemistry, in order to investigate its complex chemical reaction processes.

**Fig. 2:**
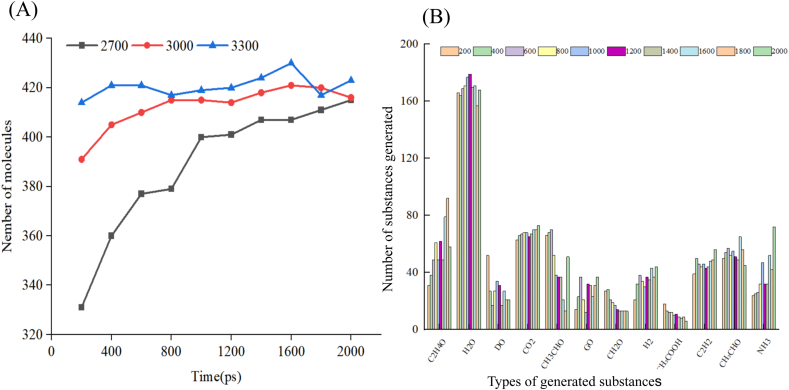
The change of the number of molecules in the pyrolysis of the Maillard product model at different temperatures (2700 K, 3000 K, 3300 K) with time (A). The changes in the type and number of compounds generated during pyrolysis at 3000 K for 2000 ps (B).

Fig. 2A shows the variation of the number of molecules with time during the pyrolysis of the MRPs model at different temperatures (2700 K, 3000 K, 3300 K). The results show that the increase of temperature will promote the increase of the number of molecules, and the growth rate is faster in the initial stage. The number of molecules continues to rise at 2700 K, which may be due to the low temperature and incomplete reaction. The number of molecules at 3000 K and 3300 K is more, indicating that the temperature contributes to the cracking of the material and increases the reaction rate. After 800 ps, the curve tends to be gentle, indicating that the reaction is basically completed. On the whole, the number of pyrolysis products at 3000 K is better, so the subsequent analysis will be based on the temperature simulation results. This conclusion is consistent with the existing research, that is, high temperature is conducive to the Maillard reaction, MRPs is more complex and the number is more (Wang et al., 2025).

Fig. 2B presents the variations in the types and quantities of compounds generated during pyrolysis at 3000 K at a reaction time of 2000 ps. In the determination of volatile substances from MRPs in hydrolyzed chicken bone extract, the components are primarily aldehydes, ketones, and heterocyclic compounds (Li et al., 2025).The intermediates obtained from pyrolysis in this study primarily include diacetyl, glyoxal, acetic acid, formaldehyde, vinyl alcohol, acetylene, acetaldehyde, and hydroxyacetaldehyde. Consistent with previous findings, as temperature increases, the time required for the formation of most products decreases, and the product composition becomes more complex (Yin et al., 2023). This phenomenon may be due to the fact that high temperature provides higher energy, accelerates the breaking of chemical bonds and reduces the activation energy of key decomposition and rearrangement reactions, which leads to a wider and faster reaction, this is consistent with previous research results (Li et al., 2025). During the pyrolysis of glutamate-glucose MRPs, a large number of gaseous products and important intermediates are generated (Kim et al., 2021). Among these, carbon dioxide, hydrogen, water, and ammonia are the dominant gaseous components. Additionally, the Amadori rearrangement products —formed during the intermediate stage of the Maillard reaction—undergo thermal degradation to generate various carbonyl compounds, with *α*-dicarbonyl compounds (*α*-DCs) being particularly prominent (Kim et al., 2021). Notably, diacetyl, methylglyoxal, and glyoxal serve as key intermediates in the formation of *α*-DCs. Previous studies on volatile compounds in fermented foods resulting from the Maillard reaction have also identified the presence of *α*-diketals, consistent with our findings. This occurrence likely stems from the fact that *α*-diketals serve as core, critical intermediate products in the degradation of sugars during the intermediate stages of the Maillard reaction. Without these compounds, the reaction cannot proceed smoothly to the final stage where flavor and color are formed (Liu et al., 2023).

### 3.3 Formation path of the key volatile substance 5-methyl-2-furanmethanol

The Lammps Reax FF software has been used to simulate the pyrolysis of coal and biomass by Reax FF MD, and has been shown to be very helpful in revealing the complex chemical reactions involved. This approach has been demonstrated to be highly effective in unraveling the intricate chemical reactions involved in such systems. Building on this, the ReaxFF MD simulation trajectories were analyzed using LAMMPS in the present study to investigate the chemical reactions occurring during the formation of MRPs.

**Fig. 3:**
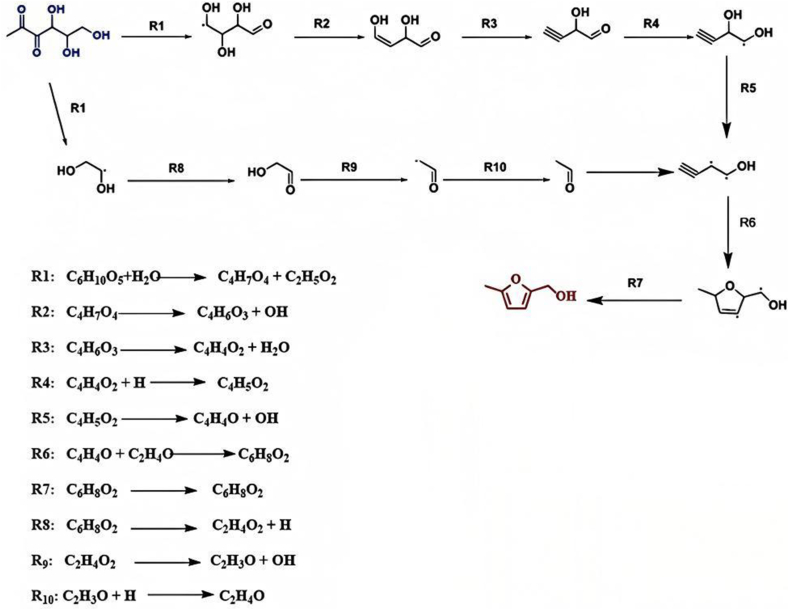
The reaction mechanism of 5-methyl-2-furanmethanol with strong aroma in the reaction model system of glutamic acid and glucose.

Fig. 3 illustrates the reaction mechanism of 5-methyl-2-furanmethanol—a compound with a distinct caramel flavor—in the glutamic acid-glucose Maillard reaction model system. This reaction pathway consists of a total of ten steps, primarily involving water molecule addition, free radical transformation, isomerization, and homolytic cleavage reactions. This pathway starts with Strecker degradation, glutamic acid reacts with glucose to produce 1-deoxyglucone, and the homolytic reaction of 1-deoxyglucone produces 3-formyl-1,2,3-trihydroxypropyl radical and 1,2-dihydroxyethyl radical. Among them, The 3-formyl-1,2,3-trihydroxypropyl radical undergoes a deprotonation reaction to form 2,4-dihydroxybut-3-enal. Then 2,4-dihydroxybut-3-enal was further dehydroxylated to form 2-hydroxybut-3-ynaldehyde. The reaction of 2-hydroxybut-3-ynaldehyde with · H produced 1,2-dihydroxybut-3-yn-1-yl radical. The 1,2-dihydroxyethyl radical can be removed by · H to form 2-hydroxyacetaldehyde. 2-Hydroxyacetaldehyde removes · OH to form formyl methyl radical. The formyl methyl radical can react with · H to form acetaldehyde. Acetaldehyde reacted with 1,2-dihydroxybut-3-yn-1-yl radical to generate 2-(hydroxy-λ3-methyl)-5-methyl-2,5-dihydrofuran-3-yl radical. Finally, 2- (hydroxy-λ3-methyl)-5-methyl-2,5-dihydrofuran-3-yl radical further undergoes free radical transfer reaction to generate 5-methyl-2-furanmethanol.

Table 3 shows the net charge distribution of 5-methyl-2-furanmethanol. Due to the electronegativity of the oxygen atom, the carbon atoms in 5-methyl-2-furanmethanol carry a lower charge. Since the molecule contains highly reactive groups such as a hydroxyl group and a furan ring, the net charge on the hydrogen atoms in the aromatic ring increases, and electrons are transferred to neighboring atoms. In the conjugated structure, the order of the C=C covalent bonds decreases, leading to reduced stability.

**Table 3:** Net charge distribution of 5-methyl-2-furanmethanol (A: atom; C: charge).

Molecular structure	A	C	A	C	A	C
5-methyl-2-furanmethanol	C (1)	−0.024	H (7)	0.134	H (13)	0.152
	C (2)	−0.177	C (8)	−0.335	H (14)	0.148
	C (3)	−0.201	H (9)	0.153	O (15)	−0.314
	C (4)	−0.111	H (10)	0.153	H (16)	0.246
	O (5)	−0.055	H11)	0.150		
	H (6)	0.121	C (12)	−0.041		

### 3.4 The search for the transition state of the MRPs 5-methyl-2-furanmethanol and the geometric structure optimization of the reactants, intermediates and products in the reaction process

Reax FF can model complex reaction processes and evolution pathways at large scales, identifying key reaction intermediates or configurations. However, they cannot explain reaction mechanisms from a molecular structural perspective. DFT enables a deeper understanding of the essence of chemical reactions by calculating molecular structures, reaction parameters, transition states, and products during chemical reactions.In transition state theory, the initial state configuration, intermediate state configurations, and final state configuration together define the complete molecular structural profile of each step within a reaction process. Among these, the molecular configuration that corresponds to the highest energy state during the intermediate stages is termed the transition state (Sun et al., 2023).

**Fig. 4:**
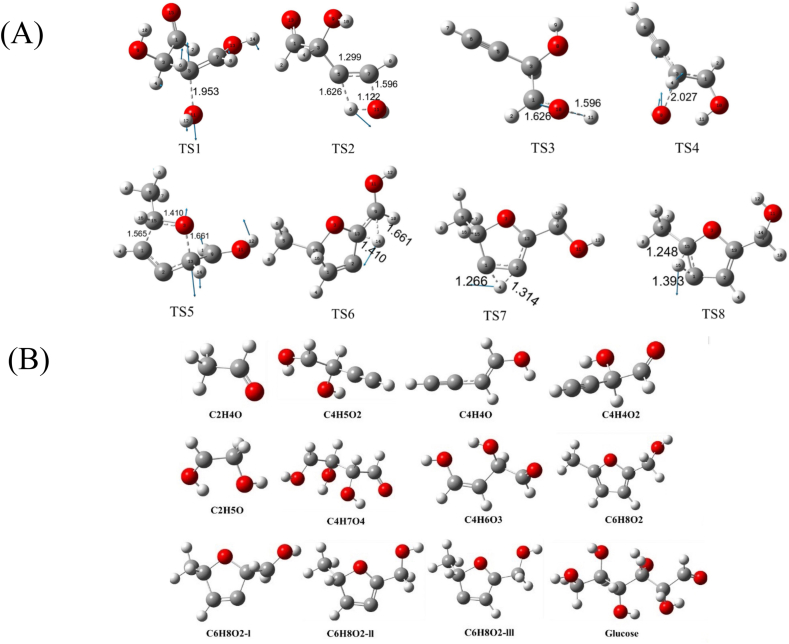
The vibration direction of the Maillard reaction product 5-methyl-2-furanmethanol reaction molecule in the virtual vibration process (A). The optimized geometries of reactants, products, intermediates and transition states (B).

Fig. 4A shows the vibration direction of the MRPs 5-methyl-2-furanmethanol reaction molecule in the virtual vibration process. As observed from this figure, the vibrational direction of the H14 atom in the transition state TS1 is consistent with that of the H6 atom in TS2. Additionally, the structure of the transition state bears similarity to that of the reactants; thus, the transition state involved in the reactant phase represents a transitional structural state analogous to the reactants. Therefore, the properties of each reaction step can be validated through vibrational analysis of the transition states.It can also be inferred from the structures and vibrational modes of the transition states that vibrational direction serves as a critical factor facilitating the conversion of transition states to products. The progression of a transition state toward the product direction can corroborate the correctness and rationality of the transition state. Specifically, the vibrational direction of TS3 is consistent with that of TS4; the vibrational direction of the H4 atom in TS4 matches that of the H11 atom in TS5; the vibrational direction of the H14 atom in TS5 aligns with that of the H14 atom in TS6; and the vibrational direction of the transition state TS7 is consistent with that of the subsequently formed TS8. In general, the vibration direction of the transition state is the same as that of the product, indicating that the configuration is reasonable and the overall reaction path is verified (Jia et al., 2021). To obtain the most stable structure of the studied system and ensure the rationality of the molecular structure, the geometric configurations of reactants, transition states, intermediates, and products were optimized at the B3LYP/6-311 (d,p) level of theory. Furthermore, the entire reaction process of 5-methyl-2-furanmethanol was contextualized and connected based on well-established reaction pathways, which facilitates a more in-depth understanding of the overall reaction mechanism. The optimized geometric configurations of the reactants, products, intermediates, and transition states are presented in Fig. 4B.

**Fig. 5:**
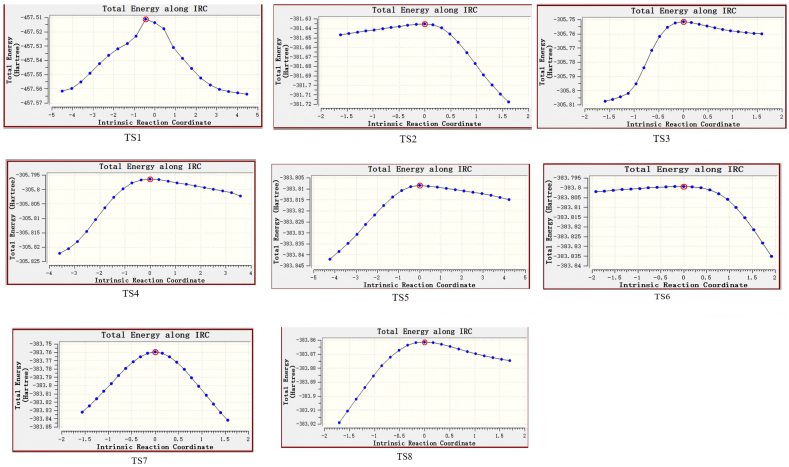
Changes in IRC energy during transient vibrations.

The energy of the transition states during the reaction also changes; Fig. 5 shows the change in IRC energy from TS1 to TS8.

### 3.5 Reaction pathway and HOMO and LUMO energy value calculations

In DFT calculation, the energy barrier diagram is used to reveal the speed and direction of chemical reaction, so as to understand the reaction mechanism and efficiency. The HOMO-LUMO orbital characterizes the electronic structure of the molecule, and its energy gap reflects the chemical stability. The orbital distribution and energy level indicate the reactivity and site. The combination of the two provides a core basis for understanding and predicting molecular properties from the energy and electronic levels, respectively.

**Fig. 6:**
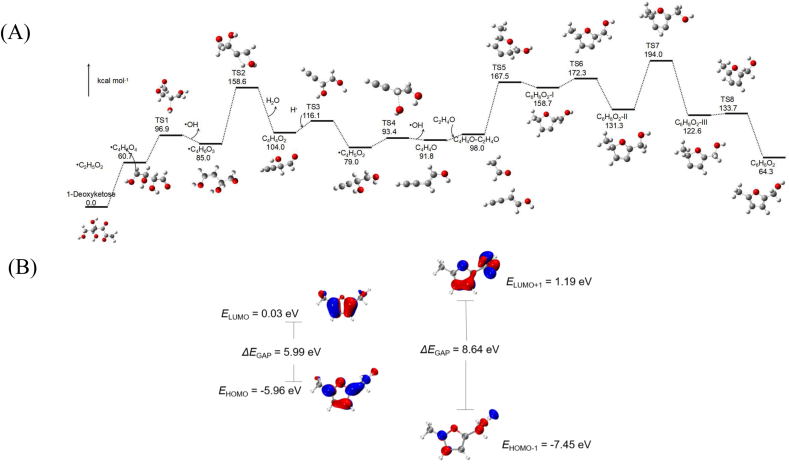
The calculated energy barrier diagram of the Maillard reaction product 5-methyl-2-furanmethanol reaction process (A). The three-dimensional HOMO, HOMO^−1^ and LUMO, LUMO ^+1^ molecular orbital energy values of 5-methyl-2-furanmethanol molecule are calculated at the B3LYP/6-311 + (d, p) level (B).

The calculated energy barrier diagram of the MRPs 5-methyl-2-furanmethanol reaction process is listed in Fig. 6A. This diagram connects all computed configurations on the potential energy surface, with all intermediates and transition states along the reaction pathway also included.1-D-deoxyglucose decomposes to yield one molecule of ·C_4_H_7_O_4_ and ·C_2_H_5_O_2_, with no transition state occurring in this process; The C-O bond cleavage of the ·C_4_H_7_O_4_ radical occurs via TS1, with a reaction energy barrier of 36.2 kcal/mol, yielding the ·C_4_H_6_O_3_ radical; The C-O bond cleavage of the ·C_4_H_6_O_3_ radical occurs via TS2, overcoming a 73.6 kcal/mol energy barrier to form C_4_H_4_O_2_ while releasing one molecule of water; ·C_4_H_4_O_2_ reacts with a H radical via TS3 to form an O-H bond, overcoming a reaction energy barrier of 12.1 kcal/mol to generate a ·C_4_H_5_O_2_ radical; The ·C_4_H_5_O_2_ radical undergoes C-O bond cleavage at transition state TS4. This step, with a reaction barrier of 14.4 kcal/mol, forms C_4_H_4_O and releases one hydroxyl radical; C_4_H_4_O and C_2_H_4_O form the complex C_4_H_4_O-C_2_H_4_O via intermolecular interaction; C_4_H_4_O-C_2_H_4_O undergoes TS5, simultaneously forming C-O and C-C bonds, overcoming a 69.5 kcal/mol energy barrier to generate the diradical molecule C_6_H_8_O_2_-I; C_6_H_8_O_2_-I undergoes TS6, undergoing intramolecular hydrogen migration, overcoming a 13.6 kcal/mol energy barrier to form C_6_H_8_O_2_-II; C_6_H_8_O_2_-II undergoes intramolecular hydrogen migration via TS7, overcoming a 62.7 kcal/mol energy barrier to form C_6_H_8_O_2_-III; C_6_H_8_O_2_-III undergoes intramolecular hydrogen migration via TS8, ultimately forming C_6_H_8_O_2_ with a final reaction energy barrier of 11.1 kcal/mol.

During the reaction, 5-methyl-2-furanmethanol undergoes energy redistribution and bond rearrangement to form the reaction transition states. However, due to the inherent instability of transition states, further bond formation and energy reconfiguration are required to generate the final products.It can be seen from the energy distribution that the energy of all transition states (TS1 to TS8) is significantly higher than that of their adjacent reactants and products, which confirms their identity as true transition states rather than stable intermediates. The highest energy barrier in the entire reaction process reaches 194.0 kcal mol^−1^, which provides direct evidence for the existence of transition states. Additionally, the reaction is clearly a multistep process, indicating that the transition states identified during the reaction correspond to genuine energy transition points within the reaction system. This observation is consistent with the vibrational characteristics of transition states reported in the preceding analysis.

Fig. 6B presents the calculated energy values of the three-dimensional HOMO, HOMO^−1^, LUMO, and LUMO^+1^ of the 5-methyl-2-furanmethanol molecule at the B3LYP/6-311+(d,p) level of theory. According to the calculation results, the band gap energy between the ground state and the first excited state of this molecule is approximately 5.99 eV,and if the molecule is stimulated by HOMO-LUMO again, the calculated value is about 8.64 eV. Due to the HOMO-LUMO energy level difference of 5.99 eV in the labeled molecule, the stability of 5-methyl-2-furan methanol was better. The bandgap between HOMO and LUMO is given by 5.99 eV, and the labeling of 5-methyl-2-furanmethanol is suitable for the chemical stability of the molecule and the transfer of electrons from the ground state to the excited state.

### 3.6 Py-GC-MS results were analyzed

**Fig. 7:**
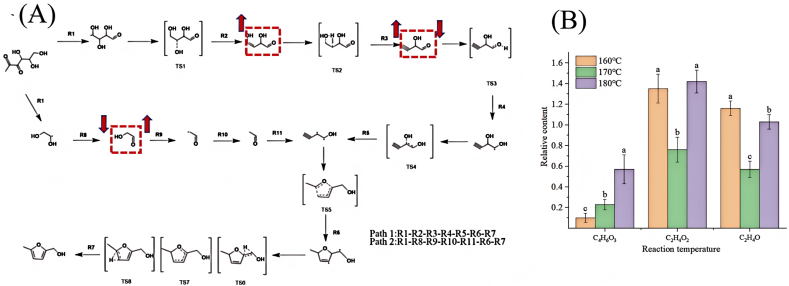
The change trend of key intermediates in the 5-methyl-2-furanmethanol pathway with temperature was obtained by combining Reax FF and DFT (A). The relative content changes of C_4_H_6_O_3_, C_2_H_4_O_2_ and C_2_H_4_O in the key substances of the Maillard reaction product (B).

To ensure consistency in experimental conditions, the samples were prepared at the same temperature used for GC-O-MS sample preparation. MRPs generated at 160 °C, 170 °C, and 180 °C were subjected to pyrolysis at 750 °C. The relative content of each component was calculated using a semi-quantitative method based on peak area. To ensure experimental reproducibility, the relative content of each component was determined in triplicate at each temperature. Subsequently, the relative standard deviation (RSD) of the relative content of each component was calculated, yielding RSD values in the range of 0.82%–7.93%. The pyrolysis products primarily consisted of volatile compounds and several cracked intermediate products. Among the volatile compounds, 1-decanol, benzyl alcohol, and 2-tridecanone were the main components, which contribute to frankincense-like and coconut-like aromas. The key intermediates included 2,4-dihydroxybut-3-enal (C_4_H_6_O_3_), 2-hydroxyacetaldehyde (C_2_H_4_O_2_), and acetaldehyde (C_2_H_4_O). Fig. 7A the change trend of key intermediates in the 5-methyl-2-furanmethanol pathway with temperature was obtained by combining Reax FF and DFT. Fig. 7B shows the relative content changes of 2,4-dihydroxybut-3-enal, 2-hydroxyacetaldehyde and acetaldehyde of the key substances in the MRPs 5-methyl-2-furanmethanol of glucose glutamate at different temperatures. The main process of sample lysis products is the decarboxylation and dehydration of the Amadori compound, which makes the C-N and C-C bonds more prone to breakage. As the sample temperature increases, the lysis product gradually becomes more complex, There are also studies that show the same trend as ours (Huang et al., 2023). According to the above findings, the pyrolysis generation process begins with residues of glucose and glutamate. Py-GC-MS results indicate that 2,4-dihydroxybut-3-enal content is low at low temperatures, with its quantity showing an upward trend as temperature increases. This trend aligns with GC-O-MS findings. This suggests that appropriately raising the temperature can promote pathway 1, likely because the low content at low temperatures indicates a higher activation energy for the reaction, requiring a certain temperature to initiate it. As temperature increases, molecular kinetic energy rises, leading to higher collision frequency and effective collision ratio. This boosts 2,4-dihydroxybut-3-enal content and accelerates pathway 1. The concentration of 2-hydroxyacetaldehyde first decreases and then increases with rising temperature, while acetaldehyde follows a similar pattern of initial decrease followed by increase. The trends for both substances align with GC-O-MS results. This may occur because, within the lower temperature range, these compounds act as intermediate products that are further consumed to form other products, leading to decreased concentrations. When temperatures exceed 170 °C, these compounds may regenerate as byproducts of certain reactions, thereby promoting pathway 2. The key intermediates identified via Py-GC-MS and their quantitative trends align with GC-O-MS results, validating the proposed pathway for 5-methyl-2-furanmethanol—a glutamic acid-glucose Maillard reaction product. This confirms that within a certain range, elevated temperatures promote pathway 1, while temperatures exceeding 170 °C favor pathway 2. It shows that the temperature has a great influence on its path, and the path is more likely to occur at higher temperatures, this is consistent with the findings of previous studies (Li et al., 2025). The key intermediates in the pathway obtained by Py-GC-MS verified the pathway of the MRPs 5-methyl-2-furanmethanol of glutamic acid-glucose proposed in this study.

## 4 Conclusions

Traditional experimental chemistry has certain limitations and cannot provide a detailed picture of the formation pathways, whereas computational chemistry can supplement these pathways. However, due to the limitations of current technical methods, the formation of free radicals in these pathways cannot be verified.This study elucidates 5-methyl-2-furanmethanol as a key caramel-flavor volatile produced in the glutamic acid–glucose Maillard reaction system by first mapping the fundamental reaction pathways with ReaxFF and then complementing these pathways with DFT calculations to reveal the intrinsic details of bond-making and bond-breaking events; In the proposed mechanism, glutamic acid–glucose initially yield 1-deoxyglucosone, which decomposes to generate radical species ·C_4_H_7_O_4_ and ·C_2_H_5_O_2_; subsequently, 5-methyl-2-furanmethanol is formed through reactions such as dehydration, dehydrogenation, and radical transfer; key intermediates identified by Py-GC–MS show trends consistent with GC–O–MS observations, supporting the proposed micro-level formation pathway for the caramel flavor compound. The combined ReaxFF-DFT approach provides a coherent microscopic mechanism and energetic rationale. Future work should quantify the relative contributions of different pathways under varying processing conditions and validate key intermediates via isotopic labeling or time-resolved spectroscopy.

## Author contributions

Xin-Pan: sample collection, experiments, writing – original draft. Chao-Kun Wei: project administration, writing – review and editing original draft, funding acquisition. Wen-Li Wang: Supervision. Ya-Nan Weng: experiments, software. An-Ran Zheng: software. Lian-Qiang Sun: conceptualization. Chun-Mei Yang: sample collection, experiments. Yuan Liu: conceptualization.

## Founding sources

This work was supported by the 10.13039/501100001809National Natural Science Foundation of China (32401996), the Fundamental Research Funds for the Central Universities (10.13039/501100012490North Minzu University, 2025QNPY10), 10.13039/501100002858China Postdoctoral Science Foundation (2024M761983), and Key research and development projects in Ningxia Hui Autonomous Region (2024BEE02034). Ningxia Hui Autonomous Region Technology Innovation Team for High-Quality Development of Characteristic Agricultural Products (2025CXTD002).

## Declaration of competing interest

The authors declare that they have no known competing financial interests or personal relationships that could have appeared to influence the work reported in this paper.

## Data Availability

Data will be made available on request.
